# Exploring the Interfacial Reaction of Nano Al/CuO Energetic Films through Thermal Analysis and Ab Initio Molecular Dynamics Simulation

**DOI:** 10.3390/molecules27113586

**Published:** 2022-06-02

**Authors:** Anran Shi, Han Zheng, Zhiyi Chen, Wei Zhang, Xiang Zhou, Carole Rossi, Ruiqi Shen, Yinghua Ye

**Affiliations:** 1 School of Chemistry and Chemical Engineering, Nanjing University of Science and Technology, Nanjing 210094, China; arshi@njust.edu.cn (A.S.); czyczyczy525@njust.edu.cn (Z.C.); zhouxiang@njust.edu.cn (X.Z.); yyinghua@mail.njust.edu.cn (Y.Y.); 2 Micro-Nano Energetic Devices Key Laboratory of MIIT, Nanjing 210094, China; 3 Institute of Space Propulsion, Nanjing University of Science and Technology, Nanjing 210094, China; 4 Tsinghua-Berkeley Shenzhen Institute (TBSI), Institute of Materials Research (iMR), Tsinghua Shenzhen International Graduate School (TSIGS), Tsinghua University, Shenzhen 518055, China; zhenghan20@mails.tsinghua.edu.cn; 5 LAAS-CNRS, University of Toulouse, 31077 Toulouse, France; rossi@laas.fr

**Keywords:** interfacial reaction, nano Al/CuO composite film, thermal analysis, ab initio molecular dynamics simulation

## Abstract

The effect of the interface layer on energy release in nanoenergetic composite films is important and challenging for the utilization of energy. Nano Al/CuO composite films with different modulation periods were prepared by magnetron sputtering and tested by differential scanning calorimetry. With the increase in the modulation period of the nano Al/CuO energetic composite films, the interface layer contained in the energetic composite film decreased meaningfully, increasing the total heat release meaningfully. Ab initio molecular dynamics (AIMD) simulation were carried out to study the preparation process changes and related properties of the nano Al/CuO energetic composite films under different configurations at 400 K. The results showed that the diffusion of oxygen atoms first occurred at the upper and lower interfaces of CuO and Al, forming AlO_x_ and Cu_x_Al_y_O_z_. The two-modulation-period structure changed more obviously than the one-modulation-period structure, and the reaction was faster. The propagation rate and reaction duration of the front end of the diffusion reaction fronts at the upper and lower interfaces were different. The Helmholtz free energy loss of the nano Al/CuO composite films with a two-modulation-period configuration was large, and the number of interfacial layers had a great influence on the Helmholtz free energy, which was consistent with the results of the thermal analysis. Current molecular dynamics studies may provide new insights into the nature and characteristics of fast thermite reactions in atomic detail.

## 1. Introduction

Nanoenergetic composite films, whose total thickness is generally 0.1–300 μm [1], consisting of alternating layers of nano-aluminum and nano-metal oxide are a new type of nano-thermite [1,2,3]. Such energetic systems of metal and metal oxides at the nanoscale are known as metastable intermolecular composites (MICs) or super-thermite [2,3,4]. MIC powder, MIC nanoenergetic composite films, MIC nanorods, and other forms are included. Composite films made of metals and metal oxides are reactive, so energetic films are called reactive multilayer films (RMFs) [5]. According to the types of chemical reactions, nanoenergetic composite films can be divided into two categories: one is metal/oxide nanoenergetic composite films that can undergo aluminothermic reactions, such as Al/CuO [3,4,6], Al/MoO_3_ [7,8], Al/Fe_2_O_3_ [9,10]_,_ and Al/NiO [11,12], etc. The other is metal/metal nanoenergetic composite films, such as Al/Ni [13,14,15], Al/Ti [16,17], and B/Ti [18,19], which can be alloyed. The volume energy density of nano Al/CuO energetic composite film is even higher than that of conventional explosive cyclotrimethyltrinitroamine (RDX). The thickness of the reactants in the nano Al/CuO energetic composite films is generally at the nanometer level, and their density can reach more than 80% of the theoretical density. The structure is orderly and uniform, and the interlayer contact area is large and close. Compared with traditional energetic materials, reactants have a high-volume energy density [20,21], fast combustion rate [22], and micron-like critical mass transfer distance [3,4]. Therefore, the heat release rate and reaction rate of energetic materials can be effectively improved. Nano Al/CuO energetic composite films are compatible with microelectromechanical systems (MEMS) [7,23] and complementary metal oxide semiconductor (COMS) manufacturing processes [24,25]. In recent years, the films have been widely used in the field of integrated energetic devices (microignition devices [25,26,27,28,29], initiation devices [30], and propulsion [31], etc.).

The fabrication of multilayered Al/CuO and Al/Ni foils and their reaction kinetics were extensively explored [32,33,34,35,36,37]. Moreover, the structure and composition of the Al/CuO interfaces were studied by experiments [5,38] and theoretical simulation [39,40]. Robert [15] et al. studied the effect of varying the bilayer spacing distribution on the reaction heat and velocity in reactive Al/Ni multilayers. Self-propagating reactions in Al/Ni nanostructured multilayer foils were examined both experimentally and computationally to determine the impact of variations in reactant spacing on reaction properties. Nicollet [41] et al. proposed an ignition model based on atomic interlayer diffusion and heat exchange. Julien [42] et al. grew Au nanoparticles in situ on the first layer of a CuO film, which showed good thermal diffusion performance, generated local hot spots, and promoted the further reaction between Al and CuO. Tichtchenko [40] et al. carried out theoretical calculations and analysis on the reaction of a fully dense layered Al/CuO system. Shen [43] et al. integrated nano Al/CuO energetic composite films into different forms of nichrome bridges by magnetron sputtering and prepared four kinds of energetic nichrome bridges with V-type angle initiators. Wang [34,37] et al. prepared Al/CuO particle laminates using a direct writing approach and found that for Al/CuO-particle-based laminates, the lateral O_2_ diffusion rate from the CuO layer to the Al layer appeared to be rate-limiting. Therefore, to explore the reaction of Al/CuO nanothermites we need to understand the interfacial layer reaction mechanisms of MICs. Researchers have studied Al/CuO, Al/Ni, and other systems, some based on macroscopic experimental phenomena or engineering applications, and some based on reaction kinetics. This paper focuses on the corresponding simulation from the atomic scale by using the AIMD method and simulates the relevant experimental phenomena at the atomic level, to find the relevant theoretical support and explanation for the decrease of energy caused by the increase of the number of interface layers.

Because the preparation process of nano Al/CuO energetic composite films is very microscopic, it is difficult to validate the preparation process by traditional experiments. Recently, high-level ab initio molecular dynamics (AIMD) has been used as an effective method to study the physical and chemical properties of nano Al/CuO energetic composite films during the preparation process [39,44]. An AIMD simulation can intuitively reflect the detailed preparation steps and the energy trends during the preparation of nano Al/CuO energetic composite films. At the same time, these results can also relate the quantitative properties of the simulations to the experimentally observed phenomena [33].

Even though so many above achievements have been achieved in the performance optimization and kinetic theory studies of nano Al/CuO energetic composite films, it is still a huge challenge to combine experiments and simulation to carry out new theoretical studies. In this work, nano Al/CuO energetic composite films with three different modulation periods were prepared by magnetron sputtering, and their thermal properties were tested by differential scanning calorimetry (DSC). The preparation process of nano Al/CuO energetic composite films at 400 K was simulated by an AIMD simulation.

## 2. Results and Discussion

### 2.1. Thermal Analysis of Nano Al/CuO Energetic Composite Films

**Figure 1:**
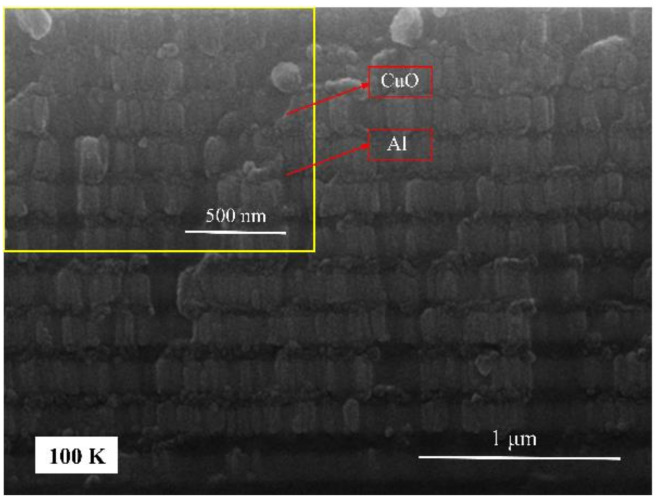
SEM cross-sectional view of nano Al/CuO composite films.

**Figure 2:**
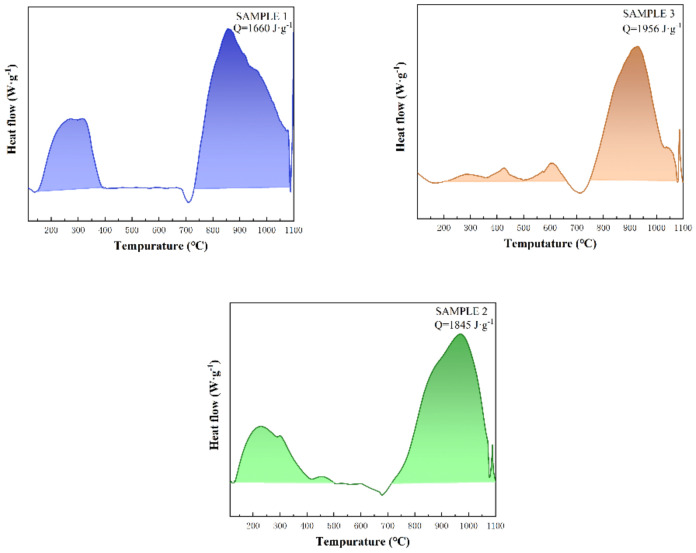
DSC curves of nano Al/CuO energetic composite films with different modulation periods.

The total thickness was kept at 3 μm with 40 modulation periods deposited as shown in Figure 1, and one modulation period was 25 nm and 50 nm thick for Al and CuO, respectively. SEM showed that the structure of the prepared films was orderly. During the preparation of nano Al/CuO energetic composite films, an interface layer is formed at the interface between the Al and CuO film. The thickness of the interface layer is generally approximately 4–15 nm, and the main components are AlO_x_ and Cu_x_Al_y_O_z_ [45]. Because the thickness of the one-modulation-period film in the nano Al/CuO energetic composite films prepared in this paper was 25 nm~150 nm, the presence of the interface layer had a significant impact on the heat release of the energetic films. In the case of the same total thickness of nano Al/CuO energetic composite films, the heat release decreased with the increase of the number of interface layers. The DSC curves of three different modulation periods of nano Al/CuO energetic composite films are shown in Figure 2, and the total heat release is shown in Table 1. The heating rate during the test was 50 K·min^−1^. With the augmentation in the modulation period of nano Al/CuO energetic composite films, the interface layer contained in the energetic composite films decreased expressively, increasing the total heat release meaningfully.

Although the total heat release of the nano Al/CuO energetic composite films rose with the increasing modulation period, the maximum value of the total heat release was only 55% of the theoretical heat release. There are three main reasons why the actual heat release of the nano Al/CuO energetic composite films is much lower than its theoretical heat release. First, the stoichiometric ratio of Al and CuO content in the energetic composite films is unbalanced. The imbalance is due to a large number of crystal defects in the preparation process of Al and CuO films, resulting in the actual density of the film being lower than its theoretical density. In addition, in the preparation of energetic composite films, not only will part of the CuO be formed into Cu_2_O due to the “O deletion phenomenon”, but part of the Al will also be oxidized to Al_2_O_3_ due to “environmental oxidation”. These results will further aggravate the imbalance of the stoichiometric ratio in energetic films. Second, the interface layer can significantly reduce the actual heat release of the energetic film, especially when the modulation period of the energetic film is equivalent to the thickness of the interface layer. As seen in Table 1, when the modulation period of the energetic film reduces from 225 nm to 75 nm, the actual heat release of the energetic composite film expressively decreases from 55% to 47%. Third, the heat loss of the test instrument increases. For traditional energetic materials, such as detonators or explosives, the temperature range of the exothermic peak of the DSC curve is only approximately 100 °C. For nano Al/CuO energetic composite films, the temperature range of the exothermic peak is approximately 400 °C. The rise in the heat release peak temperature range increases the heat loss during the test, resulting in the actual heat release from the test being lower than the theoretical value. Crystal defects and instrument heat loss are only secondary factors, and the effect of the interface layer on the heat release is the main factor.

From the above results, it can be observed that the number of interface layers of nano Al/CuO energetic composite films has a significant impact on heat release. Under the condition of the same total thickness, the smaller the modulation period of the nano Al/CuO energetic composite films, the more interface layers there are, resulting in less heat release.

### 2.2. Time Evolution of Atomic Structures

**Figure 3:**
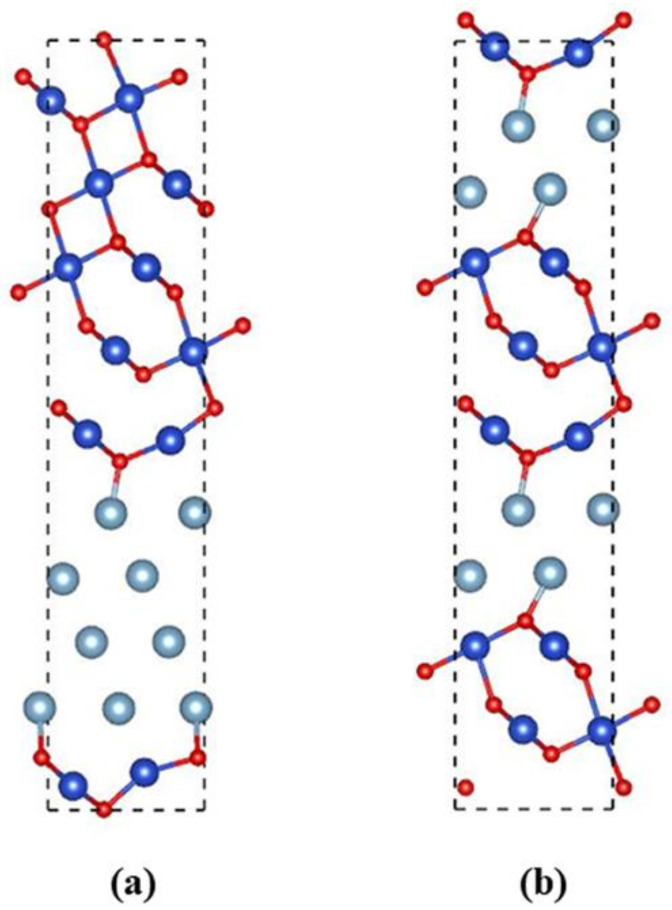
Side view of the initial configuration of (**a**) one-modulation-period structure and (**b**) two-modulation-period structure Al/CuO energetic composite films. The pale blue, blue, and red spheres represent Al, Cu, and O atoms, respectively.

The side view of the initial configuration of one-modulation-period and two-modulation-period arrays of the nano Al/CuO energetic composite films is shown in Figure 3. The simulation model involves 128 (48CuO + 32Al) atoms in a triclinic supercell.

**Figure 4:**
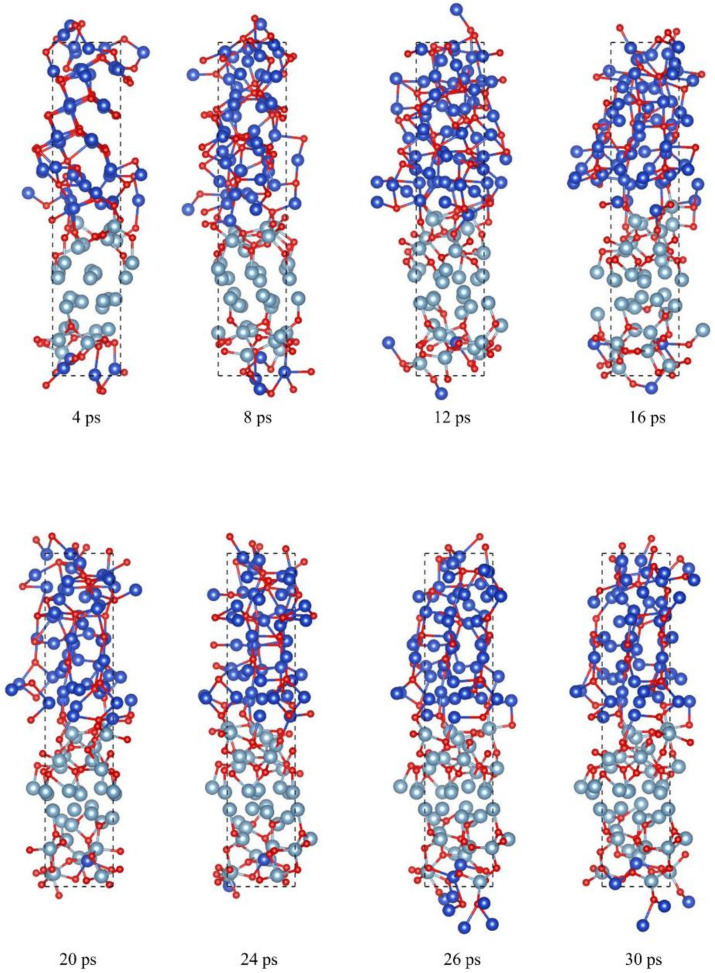
Structure changes of nano Al /CuO energetic composite films with a one-modulation-period configuration during preparation (side view). The pale blue, blue, and red spheres represent Al, Cu, and O atoms, respectively.

Figure 4 displays the vertical configuration changes of one modulation period nano Al/CuO energetic composite films with 128 atoms (48CuO + 32Al) at different times and under the same preparation conditions. The AIMD results show that the O atoms in CuO migrate to the metal Al layer, forming part of alumina, which is consistent with the experimental results and also indicates that molecular dynamics simulation can sufficiently display the experimental results of configurational changes in the Al/CuO system.

The initial reaction occurs at the interface between the CuO and Al layers. However, the initial structure of the upper interface is closer than that of the lower interface, and the interaction between the upper and lower interfaces is different at the early stage of preparation. At 4 ps, the initial reaction rate of the upper interface is faster than that of the lower interface, indicating that the symmetric structure has higher stability. When the reaction between Al and CuO progresses slowly, oxygen atoms migrate slowly to the metal Al layer and redox reaction with the Al layer near the interface but do not react with the Al in the upper two layers and the bottom three layers. A mixed layer of Al, Cu, and O atoms is formed at the interface, and the Cu atoms in the mixed layer migrate slowly to the Al layer.

**Figure 5:**
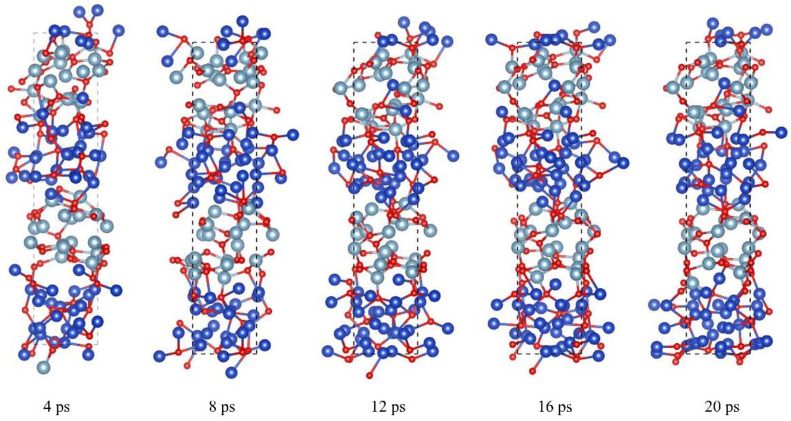
Structure changes of the nano Al /CuO energetic composite films with a two-modulation-period configuration during the preparation process (side view). The pale blue, blue, and red spheres represent Al, Cu, and O atoms, respectively.

Figure 5 shows the configuration changes in the vertical direction of the two modulation periods of Al/CuO energetic composite films with 128 atoms (48CuO + 32Al) at different times and under the same preparation conditions. The initial reaction occurs at the interface between the CuO and Al layers. However, due to the larger number of upper interface layers, the interaction between the upper and lower interfaces is different in the early preparation stage. When the reaction between Al and CuO progresses slowly, oxygen atoms migrate slowly to the metal Al layer, and the redox reaction occurs with the Al layer near the interface, forming a mixed layer of Al, Cu, and O atoms at the interface. Cu atoms in the mixed layer migrate slowly to the Al layer, and more oxygen atoms diffuse into the Al layer.

### 2.3. Reaction Front Propagation

**Figure 6:**
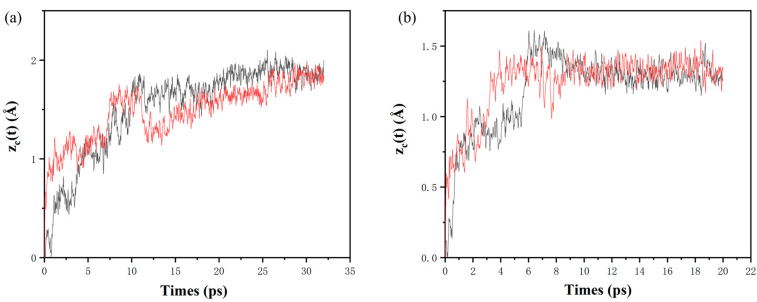
(**a**) Time evolution of the positions *z_c_(t)* of center oxygen atoms for the one-modulation-period nano Al /CuO energetic composite films. (**b**) Time evolution of the positions *z_c_(t)* of center oxygen atoms for the two-modulation-period nano Al/CuO energetic composite films.

The reaction propagation velocity can be calculated by analyzing the positional change of oxygen atoms at the interface of nano Al/CuO energetic composite films. Here, the average value of the z-axis coordinate position changes of eight O atoms at the upper and lower interfaces of the reaction front end was calculated: *Z_c_(t) = |<z_c_(t)-z_c_*(0) >|. Then, calculating the position of the moving average *<z_c_(t)>* compared with the simulated time, the propagation rate of the reaction could be obtained, and the duration of the linear propagation of the upper and lower interfaces was calculated. Table 2 lists the linear propagation rates and durations of the diffusion front ends in the nano Al/CuO energetic composite films with different configurations. Figure 6a shows that the upper and lower interfaces of nano Al/CuO energetic composite films *<z_c_(t)>* change over time.

At 400 K and with 128 atoms (48CuO + 32Al), the one-modulation-period structure of nano Al/CuO energetic composite film diffuses towards the aluminum layer within 1 ps, and *<z_c_(t)>* also enhances sharply. The oxidation front end of the upper and lower interfaces presents a linear propagation. As seen in Table 2, the propagation velocity of the upper interface (67.5 m·s^−1^) is significantly lower than that of the lower interface (440 m·s^−1^), and the duration of linear propagation are 0.432 ps and 0.140 ps, respectively. From 2 ps to 7 ps, according to the slope of *<z_c_(t)>*, the reaction speed reduces gradually. At approximately 7 ps, the movement distance of oxygen atoms at the upper and lower interfaces reaches the first peak value and then begins to oscillate, which lasts until 32 ps. Over time, the oxygen atoms in the alumina slowly move forward a short distance and interact with the metal aluminum.

When the configuration of the nano Al/CuO energetic composite film consists in 128 atoms (48CuO + 32Al) of the two-modulation-period structure, it can be seen from Figure 6b that the propagation velocity of the lower interface is always greater than that of the upper interface. This is due to the difference in the tightness of the upper and lower interfaces. Compared with the one-modulation-period structure of Al/CuO energetic composite films, the upper interface propagation rate (182.1 m·s^−1^) and the lower interface propagation rate (681.7 m·s^−1^) are higher. The durations of linear propagation at the upper and lower interfaces are 0.067 ps and 0.0875 ps, respectively. The propagation rate of the upper and lower interfaces varies from 1 ps to 6 ps, *<z_c_(t)>*, with very little difference after 6 ps. The trend of the upper and lower interface velocity can provide a corresponding basis for our subsequent judgment of energy loss

### 2.4. Change in the Helmholtz Free Energy during Preparation

**Figure 7:**
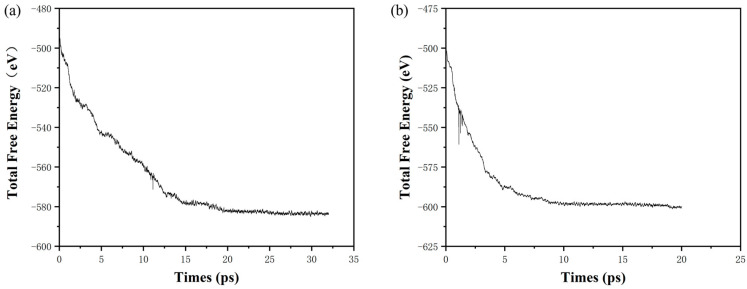
(**a**) Change of Helmholtz free energy of nano Al/CuO energetic composite films with a one-modulation-period configuration during the preparation process. (**b**) Change of free energy of Helmholtz nano Al/CuO energetic composite films with a two-modulation-period configuration during the preparation process.

During the simulation, the temperature was 400 K. According to the second law of thermodynamics, in the isothermal process, the maximum work that a closed system can do is equal to the reduction in its Helmholtz free energy, which is the ability of the system to do work under isothermal conditions. As shown in Figure 7a, the initial Helmholtz free energy of the one-modulation-period nano Al/CuO energetic composite films with 128 atoms (48CuO + 32Al) is −500.42 eV. After stabilizing, the Helmholtz free energy is −583.00 eV. The Helmholtz free energy consumed in the process is approximately 80.00 eV.

As shown in Figure 7b, the Helmholtz free energy of the 128-atom (48CuO + 32Al) nano Al/CuO energetic composite film configuration in the two-modulation-period arrangement is −503.60 eV. After stabilizing, the Helmholtz free energy is −600.00 eV. The Helmholtz free energy consumed in the process is approximately −100.00 eV. According to the above simulation data, the one-modulation-period structure Al/CuO energetic composite films consume more Helmholtz free energy, and the state is more chaotic than that of the two-modulation-period structure Al/CuO energetic composite films when it tends to be stable. This result indicates that part of the energy has been consumed in the preparation and storage process of the sample, and the energy consumption value rises with the change in configuration and the increase in the number of interface layers.

Based on the results calculated by the AIMD simulation and experiment results of thermal analysis, it can be found that in the preparation process, the formation of oxygen diffusion and compound only happens near the interface layer, as a result of the two-modulation-period configuration containing nano Al/CuO energetic composite films that have more interface layers; the preparation process of the configuration change is more complicated, since the higher propagation rate of oxygen atoms in the upper and lower interfaces leads to more Helmholtz free energy loss in the preparation process. This is due to the formation of AlO_x_ and Cu_x_Al_y_O_z_ near the interface layer, which is consistent with the phenomenon that the cross-section image taken by SEM cannot get clear boundaries. In the AIMD simulation process, we presented the changing trend of Helmholtz free energy. As the number of interface layers increases, the more Helmholtz free energy is lost in the preparation process, the lower the initial energy is, and the less heat is released in the thermal analysis process, which is consistent with the thermal analysis results and the experimental results of the thermal analysis.

## 3. Experimental Section

### 3.1. Preparation of Nano Al/CuO Energetic Composite Films

Nano Al/CuO energetic composite films were prepared by a magnetron sputtering coater (MS550, Nanjing University of Science and Technology). The Al target (diameter: 76 mm; thickness: 5 mm; purity: 99.99%, Zhongnuo New Material Technology Co., Ltd.) and CuO target (diameter: 76 mm; thickness: 4 mm; purity: 99.99%, Zhongnuo New Material Technology Co., Ltd.) were placed symmetrically at the target head position at the bottom of the vacuum chamber. The Al target was sputter-coated by direct current (DC) magnetron sputtering with a power of 150 W and a sputtering rate of 50 nm/min. Radio frequency (RF) magnetron sputtering was used for the CuO target, the sputtering power was 200 W and the sputtering rate was 27 nm/min. Before magnetron sputtering began, the pressure in the vacuum chamber was evacuated to 9 × 10^−4^ Pa, the vacuum gauge was set manually, and high-purity argon (99.99%) was pumped with a flow rate of 12 sccm to maintain the working pressure at 0.4 Pa. At this point, the Al target was first illuminated, then the CuO target was illuminated, and the time to switch the baffle above the target was set by a computer to adjust the thickness of film deposition. The substrate of the film sample was placed on a rotating sample platform above the vacuum chamber, and the multilayer films were prepared by repeatedly rotating between the Al target and CuO target.

The aluminothermic reaction between Al and the CuO film are as follows:

2*Al* + 3*CuO* = *Al*_2_*O*_3_ + 3*Cu* (*Q*_1_ = −4070 J·g^−1^)

2*Al* + 3*Cu*_2_*O* = *Al*_2_*O*_3_ + 6*Cu* (*Q*_2_ = −2400 J·g^−1^)

The density of the film was approximately that of the target material (Al: 2.7 g·cm^−3^, CuO: 6.0 g·cm^−3^). According to the coating parameters, the modulation ratio of Al and CuO in the prepared nano Al/CuO energetic composite films was 1:2, and the chemical molar mass ratio of Al and CuO in the nano Al/CuO energetic composite films was 2:3, which was consistent with the theoretical stoichiometric ratio of the reaction equation. Therefore, the oxygen equilibrium state of the reaction system composed of the Al/CuO energetic film was zero, and the reaction heat of formation of the reaction system was calculated to be −3569 J·g^−1^. The sample parameters of the nano Al/CuO energetic composite films with different modulation periods are shown in Table 3. In this paper, the combination of the single-layer aluminum film and the single-layer copper oxide film was regarded as a modulation period.

### 3.2. Thermal Property Analysis

The heat releases of nano Al/CuO energetic composite films were recorded from 100 °C to 1100 °C using DSC (NETZSCH STA 449C synchronous thermal analyzer, Germany) at a heating rate of 50 K·min^−1^ under a 30 mL·min^−1^ 99.999% argon flow.

### 3.3. Computational Methodology

The preparation of nano Al/CuO energetic composite films under certain conditions was simulated by AIMD, and the stoichiometric ratio was 2:3 for the perfect reaction under ideal conditions. In the early stage, a K-type thermocouple was used to test the temperature of the substrate. The test results showed that in the preparation process, the stable temperature of the sample was approximately 75 °C when the Al target was sputtered by a 150 W DC source, and the stable temperature of the sample was approximately 230 °C when the CuO target was sputtered by 200 W RF source. After comprehensive consideration, 400 K was selected as the simulation temperature of AIMD.

To study the reactivity of the interfacial layer of nano Al/CuO energetic composite films, an AIMD simulation within the framework of spin-polarized density functional(DFT) theory [46,47] was performed using the Vienna Simulation Package(VASP) [48,49,50]. The Kohn–Sham equations were solved with a plane-wave basis set and periodic boundary conditions. Electron exchange and correlation were described using the Perdew–Burke–Ernzerhof(PBE) [51] version of the generalized gradient approximation (GGA). The ion cores were described by projector-augmented wave (PAW) [52] potential as implemented by Kresse and Joubert [53]. The on-site Coulomb repulsion (Hubbard U) was applied to the Cu d state, where *U* = 7.5 eV and *J* = 0.98 eV [54]. The Monkhorst–Pack scheme [55] was used to sample K points in the Brillouin zone, and the grid of K points was 1 × 1 × 1. We utilized an energy cutoff of 500 eV in the plane-wave basis set expansion for both geometry optimization and molecular dynamics calculations. During the geometry relaxation, the total energy was converged up to 10^−4^ eV/atom using Gaussian smearing with a smearing width of 0.1 eV, gamma k-points for the sampling of the Brillouin zone, and the convergence standard of the interatomic interaction force was 0.05 eV/Å. In the process of kinetic simulation, the convergence standard of single-atom energy was 10^−5^ eV. We relaxed all atomic positions (no constraints) according to their forces using a conjugate gradient algorithm and the relaxation stopped when the force on each atom was below 0.1 eV/Å.

The simulation model with 128 (48CuO + 32Al) atoms was established to verify the effect of one modulation period and two modulation periods Al/CuO arrangements on the stability of the sample under the condition of the same atomic number. In the process of kinetic simulation, the convergence standard of single-atom energy was 10^−5^ eV, and 1 × 2 × 1 triclinic supercells (*L_x_*, *L_y_*, *L_z_* = 11.5 Å, 5.8 Å, 26.3 Å and *β* = 66.0°) were constructed. The canonical ensemble (NVT) system was used for AIMD simulation. A Nosè–Hoover hot bath [56,57] was used for temperature control with a time step of 0.8 fs and a simulation time of 20 ps for the two-modulation-period model. The one-modulation-period model had a longer convergence time than the two-modulation-period model and the simulation time was 32 ps.

## 4. Conclusions

In summary, we prepared nano Al/CuO energetic composite films with the same total thickness and different modulation periods, studied their thermal properties, and analyzed their actual heat release. The experimental results show that the number of interface layers of nano Al/CuO energetic composite films has a very obvious effect on the heat release. The smaller the modulation period of the nano Al/CuO energetic composite films, the greater the number of interfacial layers and the lower the heat release. AIMD was used to simulate the preparation process and properties of nano Al/CuO energetic composite films under different configurations at 400 K. The results show that the diffusion of oxygen atoms first occurred at the upper and lower interfaces of CuO and Al, forming AlO_x_ and Cu_x_Al_y_O_z_. The two-modulation-period structure changed more obviously than the one-modulation-period structure, and the reaction was faster with a duration of more than 1 ps. The change in Helmholtz free energy of the one-modulation-period Al/CuO energetic composite films was less than that of the two-modulation-period structure Al/CuO energetic composite films, indicating that the two-modulation-period structure Al/CuO energetic composite films lost more Helmholtz free energy than the one modulation period structure during the preparation process. The number of interfacial layers had a great influence on the Helmholtz free energy in the preparation process, the two-modulation-period structure Al/CuO energetic composite films lost more energy, and the lower the initial energy was, the less heat was released in the thermal analysis process, which was consistent with the AIMD simulations results and the thermal analysis experimental results. This mechanism may guide us to tailor the reactivity of the interface layer by controlling the transport rate of the oxygen atoms. Comparing the phenomena of the DSC experimental test with AIMD simulation results, we could get the reason why the number of interface layers affects the subsequent energy in the preparation process. AIMD studies have explained the interfacial diffusion properties of nano Al/CuO energetic composite films at the atomic level, which may provide new insights into understanding the reactive properties in atomic details and help improve the interface reaction in manufacturing.

## Figures and Tables

**Table 1:** Comparison of heat release of nano Al /CuO energetic composite films with different modulation periods.

Sample Number	Modulation Period /nm	Number of Interface Layers	DSC Heat Quantity /(J·g^−^^1^)	DSC Heat Release/Theoretical Heat Release
SAMPLE1	75	79	1660	47%
SAMPLE2	150	39	1845	52%
SAMPLE3	225	25	1956	55%

**Table 2:** Linear propagation rates and durations of the upper and lower diffusion fronts of nano Al/CuO energetic composite films with different configurations.

	Upper Interface		Lower Interface	
	Velocity (m/s)	Time of Duration (ps)	Velocity (m/s)	Time of Duration (ps)
One-modulation-period structure	67.5	0.4320	440.0	0.1400
Two-modulation-period structure	182.1	0.0670	681.7	0.0875

**Table 3:** Sample parameters of nano Al /CuO energetic composite films with different modulation periods.

Sample Number	Modulation Period /nm	Single Al Thickness /nm	Single CuO Thickness /nm	Number of Modulation Period	Total Thickness /nm
SAMPLE1	75	25	50	40	3000
SAMPLE2	150	50	100	20	3000
SAMPLE3	225	75	150	13	3000

## Data Availability

Not applicable.

## Sample Availability

Samples of the compounds are not available from the authors.

## References

[1] Adams D.P. (2015). Reactive multilayers fabricated by vapor deposition: A critical review. Thin Solid Film..

[2] Rossi C., Zhang K., Esteve D., Alphonse P., Tailhades P., Vahlas C. (2007). Nanoenergetic Materials for MEMS: A Review. J. Microelectromechanical Syst..

[3] Zhou X., Torabi M., Lu J., Shen R.Q., Zhang K.L. (2014). Nanostructured Energetic Composites: Synthesis, Ignition/Combustion Modeling, and Applications. ACS Appl. Mater. Interfaces.

[4] He W., Liu P.J., He G.Q., Gozin M., Yan Q.L. (2018). Highly Reactive Metastable Intermixed Composites (MICs): Preparation and Characterization. Adv. Mater..

[5] Salvagnac L., Assie-Souleille S., Rossi C. (2020). Layered Al/CuO Thin Films for Tunable Ignition and Actuations. Nanomaterials.

[6] Rossi C. (2014). Two Decades of Research on Nano-Energetic Materials. Propellants Explos. Pyrotech..

[7] Tai Y., Xu J.B., Wang F., Dai J., Zhang W., Ye Y.H., Shen R.Q. (2018). Experimental and modeling investigation on the self-propagating combustion behavior of Al-MoO3 reactive multilayer films. J. Appl. Phys..

[8] Dutro G.M., Yetter R.A., Risha G.A., Son S.F. (2009). The effect of stoichiometry on the combustion behavior of a nanoscale Al/MoO(3) thermite. Proc. Combust. Inst..

[9] Zhou X., Zhu Y., Ke X., Zhang K.L. (2019). Exploring the solid-state interfacial reaction of Al/Fe2O3 nanothermites by thermal analysis. J. Mater. Sci..

[10] Zhang W.C., Yin B.Q., Shen R.Q., Ye J.H., Thomas J.A., Chao Y.M. (2013). Significantly Enhanced Energy Output from 3D Ordered Macroporous Structured Fe2O3/Al Nanothermite Film. ACS Appl. Mater. Interfaces.

[11] Firat Y.E. (2020). Influence of current density on Al:NiO thin films via electrochemical deposition: Semiconducting and electrochromic properties. Mat. Sci. Semicon. Proc..

[12] Yan Y.C., Shi W., Jiang H.C., Xiong J., Zhang W.L., Li Y.R. (2015). Fabrication and Characterization of Al/NiO Energetic Nanomultilayers. J. Nanomater..

[13] Baras F., Turlo V., Politano O., Vadchenko S.G., Rogachev A.S., Mukasyan A.S. (2018). SHS in Ni/Al Nanofoils: A Review of Experiments and Molecular Dynamics Simulations. Adv. Eng. Mater..

[14] Dreizin E.L. (2009). Metal-based reactive nanomaterials. Prog. Energy Combust. Sci..

[15] Knepper R., Snyder M.R., Fritz G., Fisher K., Knio O.M., Weihs T.P. (2009). Effect of varying bilayer spacing distribution on reaction heat and velocity in reactive Al/Ni multilayers. J. Appl. Phys..

[16] Fu K.K., Sheppard L., Chang L., An X.H., Yang C.H., Ye L. (2018). Comparative study on plasticity and fracture behaviour of Ti/Al multilayers. Tribol. Int..

[17] Sen S., Lake M., Wilden J., Schaaf P. (2017). Synthesis and characterization of Ti/Al reactive multilayer films with various molar ratios. Thin Solid Film..

[18] Zhang Y.X., Wang Y., Ai M.T., Jiang H.C., Yan Y.C., Zhao X.H., Wang L., Zhang W.L., Li Y.R. (2018). Reactive B/Ti Nano-Multilayers with Superior Performance in Plasma Generation. ACS Appl. Mater. Interfaces.

[19] Reeves R.V., Rodriguez M.A., Jones E.D., Adams D.P. (2012). Condensed-Phase and Oxidation Reaction Behavior of Ti/2B Foils in Varied Gaseous Environments. J. Phys. Chem. C.

[20] Blobaum K.J., Reiss M.E., Plitzko J.M., Weihs T.P. (2003). Deposition and characterization of a self-propagating CuOx/Al thermite reaction in a multilayer foil geometry. J. Appl. Phys..

[21] Manesh N.A., Basu S., Kumar R. (2010). Experimental flame speed in multi-layered nano-energetic materials. Combust. Flame.

[22] Kwon J., Ducere J.M., Alphonse P., Bahrami M., Petrantoni M., Veyan J.F., Tenailleau C., Esteve A., Rossi C., Chabal Y.J. (2013). Interfacial chemistry in Al/CuO reactive nanomaterial and its role in exothermic reaction. ACS Appl. Mater. Interfaces.

[23] Xu J., Tai Y., Ru C., Dai J., Ye Y., Shen R., Zhu P. (2017). Tuning the Ignition Performance of a Microchip Initiator by Integrating Various Al/MoO3 Reactive Multilayer Films on a Semiconductor Bridge. ACS Appl Mater Interfaces.

[24] Azadmanjiri J., Berndt C.C., Wang J., Kapoora A., Srivastava V.K. (2016). Nanolaminated composite materials: Structure, interface role and applications. RSC Adv..

[25] Taton G., Lagrange D., Conedera V., Renaud L., Rossi C. (2013). Micro-chip initiator realized by integrating Al/CuO multilayer nanothermite on polymeric membrane. J. Micromechanics Microengineering.

[26] Zhu P., Shen R., Ye Y., Fu S., Li D. (2013). Characterization of Al/CuO nanoenergetic multilayer films integrated with semiconductor bridge for initiator applications. J. Appl. Phys..

[27] Ahn J.Y., Kim S.B., Kim J.H., Jang N.S., Kim D.H., Lee H.W., Kim J.M., Kim S.H. (2016). A micro-chip initiator with controlled combustion reactivity realized by integrating Al/CuO nanothermite composites on a microhotplate platform. J. Micromechanics Microengineering.

[28] Yan Y., Shi W., Jiang H., Xiong J., Zhang W., Li Y. (2015). Characteristics of the Energetic Igniters Through Integrating Al/NiO Nanolaminates on Cr Film Bridge. Nanoscale Res. Lett..

[29] Yan Y., Shi W., Jiang H., Cai X., Deng X., Xiong J., Zhang W. (2015). Characteristics of the Energetic Igniters Through Integrating B/Ti Nano-Multilayers on TaN Film Bridge. Nanoscale Res. Lett..

[30] Zhou X., Shen R.Q., Ye Y.H., Zhu P., Hu Y., Wu L.Z. (2011). Influence of Al/CuO reactive multilayer films additives on exploding foil initiator. J. Appl. Phys..

[31] Glavier L., Taton G., Ducéré J.-M., Baijot V., Pinon S., Calais T., Estève A., Djafari Rouhani M., Rossi C. (2015). Nanoenergetics as pressure generator for nontoxic impact primers: Comparison of Al/Bi2O3, Al/CuO, Al/MoO3 nanothermites and Al/PTFE. Combust. Flame.

[32] Fu S., Shen R., Zhu P., Ye Y. (2019). Metal–interlayer–metal structured initiator containing Al/CuO reactive multilayer films that exhibits improved ignition properties. Sens. Actuators A Phys..

[33] Shi A., Zhang W., Shen R. (2021). Self-propagating combustion simulation of sputter-deposited nano-energetic multilayer films. J. Phys. Conf. Ser..

[34] Wang H., Julien B., Kline D.J., Alibay Z., Rehwoldt M.C., Rossi C., Zachariah M.R. (2020). Probing the Reaction Zone of Nanolaminates at ∼μs Time and ∼μm Spatial Resolution. J. Phys. Chem. C.

[35] Esteve A., Lahiner G., Julien B., Vivies S., Richard N., Rossi C. (2020). How Thermal Aging Affects Ignition and Combustion Properties of Reactive Al/CuO Nanolaminates: A Joint Theoretical/Experimental Study. Nanomaterials.

[36] Brotman S., Rouhani M.D., Rossi C., Estève A. (2019). A condensed phase model of the initial Al/CuO reaction stage to interpret experimental findings. J. Appl. Phys..

[37] Wang H., Biswas P., Zachariah M.R. (2022). Direct Imaging and Simulation of the Interface Reaction of Metal/Metal Oxide Nanoparticle Laminates. J. Phys. Chem. C.

[38] Rossi C. (2019). Engineering of Al/CuO Reactive Multilayer Thin Films for Tunable Initiation and Actuation. Propell. Explos. Pyrot..

[39] Xiong G., Yang C., Feng S., Zhu W. (2020). Ab initio molecular dynamics studies on the transport mechanisms of oxygen atoms in the adiabatic reaction of Al/CuO nanothermite. Chem. Phys. Lett..

[40] Tichtchenko E., Esteve A., Rossi C. (2021). Modeling the self-propagation reaction in heterogeneous and dense media: Application to Al/CuO thermite. Combust. Flame.

[41] Nicollet A., Lahiner G., Belisario A., Souleille S., Djafari-Rouhani M., Estève A., Rossi C. (2017). Investigation of Al/CuO multilayered thermite ignition. J. Appl. Phys..

[42] Julien B., Cure J., Salvagnac L., Josse C., Esteve A., Rossi C. (2020). Integration of Gold Nanoparticles to Modulate the Ignitability of Nanothermite Films. ACS Appl. Nano Mater..

[43] Shen Y., Xu J., Wang C., Yang T., Ye Y., Shen R. (2020). Ignition characteristics of energetic nichrome bridge initiator based on Al/CuO reactive multilayer films under capacitor discharge and constant current conditions. Sens. Actuators A Phys..

[44] Wu Q., Xiong G., Zhu W., Xiao H. (2015). How does low temperature coupled with different pressures affect initiation mechanisms and subsequent decompositions in nitramine explosive HMX?. Phys. Chem. Chem. Phys..

[45] Abdallah I., Zapata J., Lahiner G., Warot-Fonrose B., Cure J., Chabal Y., Esteve A., Rossi C. (2018). Structure and Chemical Characterization at the Atomic Level of Reactions in Al/CuO Multilayers. ACS Appl. Energy Mater..

[46] Hohenberg P., Kohn W. (1964). Inhomogeneous Electron Gas. Phys. Rev..

[47] Kohn W., Sham L.J. (1965). Self-Consistent Equations Including Exchange and Correlation Effects. Phys. Rev..

[48] Kresse G., Hafner J. (1993). Ab initio molecular dynamics for open-shell transition metals. Phys. Rev. B Condens. Matter..

[49] Kresse G., Furthmüller J. (1996). Efficiency of ab-initio total energy calculations for metals and semiconductors using a plane-wave basis set. Comput. Mater. Sci..

[50] Kresse G., Furthmüller J. (1996). Efficient Iterative Schemes for Ab Initio Total-Energy Calculations Using a Plane-Wave Basis Set. Phys. Rev. B.

[51] Perdew J.P., Burke K., Ernzerhof M. (1996). Generalized Gradient Approximation Made Simple. Phys. Rev. Lett..

[52] Blochl P.E. (1994). Projector augmented-wave method. Phys. Rev. B Condens. Matter..

[53] Kresse G., Joubert D. (1999). From ultrasoft pseudopotentials to the projector augmented-wave method. Phys. Rev. B.

[54] Anisimov V.I., Zaanen J., Andersen O.K. (1991). Condensed matter. Band Theory and Mott Insulators: Hubbard U Instead of Stoner I. Phys. Rev. B.

[55] Monkhorst H.J., Pack J.D. (1976). Special points for Brillouin-zone integrations. Phys. Rev. B.

[56] Nosé S. (1984). A Molecular Dynamics Method for Simulations in the Canonical Ensemble. Mol. Phys..

[57] Hoover W.G. (1985). Canonical dynamics: Equilibrium phase-space distributions. Phys. Rev. A.

